# Age Prediction Using DNA Methylation Heterogeneity Metrics

**DOI:** 10.3390/ijms25094967

**Published:** 2024-05-02

**Authors:** Dmitry I. Karetnikov, Stanislav E. Romanov, Vladimir P. Baklaushev, Petr P. Laktionov

**Affiliations:** 1 Federal Research Center Institute of Cytology and Genetics SB RAS, 630090 Novosibirsk, Russia; 2 Epigenetics Laboratory, Department of Natural Sciences, Novosibirsk State University, 630090 Novosibirsk, Russia; 3 Institute of Molecular and Cellular Biology, Siberian Branch of the Russian Academy of Sciences, 630090 Novosibirsk, Russia; 4 Federal Center for Brain and Neurotechnologies, Federal Medical and Biological Agency of Russia, 117513 Moscow, Russia; 5 Engelhardt Institute of Molecular Biology, Russian Academy of Sciences, 119991 Moscow, Russia; 6 Department of Medical Nanobiotechnology, Medical and Biological Faculty, Pirogov Russian National Research Medical University, Ministry of Health of the Russian Federation, 117997 Moscow, Russia

**Keywords:** DNA methylation heterogeneity, epigenetic age, bisulfite sequencing, eAge clocks

## Abstract

Dynamic changes in genomic DNA methylation patterns govern the epigenetic developmental programs and accompany the organism‘s aging. Epigenetic clock (eAge) algorithms utilize DNA methylation to estimate the age and risk factors for diseases as well as analyze the impact of various interventions. High-throughput bisulfite sequencing methods, such as reduced-representation bisulfite sequencing (RRBS) or whole genome bisulfite sequencing (WGBS), provide an opportunity to identify the genomic regions of disordered or heterogeneous DNA methylation, which might be associated with cell-type heterogeneity, DNA methylation erosion, and allele-specific methylation. We systematically evaluated the applicability of five scores assessing the variability of methylation patterns by evaluating within-sample heterogeneity (WSH) to construct human blood epigenetic clock models using RRBS data. The best performance was demonstrated by the model based on a metric designed to assess DNA methylation erosion with an MAE of 3.686 years. We also trained a prediction model that uses the average methylation level over genomic regions. Although this region-based model was relatively more efficient than the WSH-based model, the latter required the analysis of just a few short genomic regions and, therefore, could be a useful tool to design a reduced epigenetic clock that is analyzed by targeted next-generation sequencing.

## 1. Introduction

DNA methylation is considered a key epigenetic mark that plays a role in the regulation of gene expression, chromatin functions, genome stability, and spatial nuclear architecture [1]. Since DNA methylation dynamics accompany normal development and pathological processes, specific methylation patterns may serve as hallmarks of different cell types in their particular state [2,3,4]. Therefore, with the development of high-throughput sequencing and methylation microarrays, the analysis of DNA methylation is currently widely used for disease diagnostics as well as the assessment of health risks and aging [5,6,7]. More specifically, algorithms of epigenetic age (eAge) clocks analyze DNA methylation to predict chronological and biological age, and they serve as powerful tools for the assessment of various age-related health risks, including diseases, mental disorders, and all-cause mortality [2,8].

There are currently a few types of eAge clocks that have been developed for humans, mice, and other animals [7]. Most human eAge clocks are based on the regression modeling of microarray data that provides information about the individual methylation levels for thousands of predefined CpGs simultaneously [7]. While initial models predicted age by several hundreds of CpGs, the detailed analysis of high-throughput methylation arrays enables researchers to restrict the list of diagnostically relevant CpGs to a few items, thereby making it possible to analyze samples with cost-effective real-time PCR, SnapShot, pyrosequencing, or targeted bisulfite sequencing (BS-seq) methods [7].

Although microarrays are powerful tools for developing methylation clocks, this technology has several drawbacks. First, widespread commercial high-throughput human microarrays are limited to predefined sets of up to 860,000 CpGs that cover around 3% of all CpG sites in the human genome [9]. Second, microarray analysis measures the methylation level of each CpG independently and is unable to extract contiguous patterns of DNA methylation as they are considered functional regulatory units [10]. These particular limitations can be overcome by using whole-genome or reduced representation bisulfite sequencing (WGBS or RRBS, respectively) methods that are based on the next-generation short-read sequencing of randomly fragmented bisulfite-converted genome DNA [11]. Specifically, WGBS covers around 90% of CpGs, and RRBS enables the analysis of around 10–20% of CpG sites, depending on the sequencing depth [12]. The main disadvantage of RRBS and WGBS is the non-uniform CpG-site coverage across samples due to the difference in sequencing depth and library preparation. The resulting inter-sample variation can be a source of uncertainty when a model built on one dataset is applied to separate samples [13]. Microarray DNA methylation data are considered standardized in this regard. However, WGBS and RRBS make it possible to analyze contiguous methylation patterns, methylation haplotype blocks, and methylation heterogeneity. Given these advantages, these methods are now widely used to discriminate cell types, and they have proven to be beneficial for cancer research and “liquid biopsy” diagnostics using circulating plasma DNA samples [5,10,14,15]. However, WGBS and RRBS are rarely used for eAge clock development and analysis [13].

The data obtained with WGBS and RRBS, to some extent, are quite difficult to interpret because it does not simply aggregate the average methylation levels of individual CpGs across all cells like in DNA methylation microarrays; instead, they reflect sample-specific sequential methylation patterns with single-molecule resolution. However, since it is believed that reproducible aging-related dynamics in DNA methylation might be caused by changes in organ cell composition due to differentiation, stem, and somatic cell loss or by epigenetic drift [2,16,17,18], such high-resolution data could potentially improve eAge clock development. Nevertheless, the first step when building a model using sequencing data is to bring continuous methylation patterns from every individual DNA molecule to the array of scores. This straightforward solution, which relies on the aggregation of the average methylation levels of individual CpGs within overlapping reads in the manner of microarray data, has been used previously for constructing RRBS-based eAge models [13,19]. However, this approach completely loses information about the sample-specific diversity of methylation patterns.

Alternatively, one can use a set of numerical metrics called within-sample heterogeneity scores (WSH) that capture different sources of DNA methylation heterogeneity, such as cell-type composition, allele-specific DNA methylation, or DNA methylation erosion, for every single genome region [10,12,20,21]. However, until recently, it was not clear whether such metrics were suitable for estimating chronological age since, strictly speaking, they are not directly related to the total CpG methylation level. Nevertheless, the novel results obtained on the mouse RRBS data showed that the dynamics of methylation patterns, as reflected in the form of the epigenetic “disorder” criteria, are suitable for the construction of the region-based epigenetic clocks [22]. In the present paper, we wanted to examine the ability of different heterogeneity measures to predict chronological age using the RRBS dataset from human whole blood DNA.

Here, we proposed an algorithm for epigenetic clock design based on the analysis of DNA methylation heterogeneity patterns rather than the methylation level. To identify genome regions showing an age-correlated increase or decrease in heterogeneity, we applied the previously described WSH metrics: MHL (Methylation Haplotype Load), PDR (Proportion of Discordant Reads), PM (Epipolymorphism), FDRP (Fraction of Discordant Read Pairs), and qFDRP (quantitative Fraction of Discordant Read Pairs) [12]. Genomic regions showing the strongest association between methylation heterogeneity and age were associated with genes related to aging or linked with CpGs that are included in different epigenetic age models. We built five epigenetic clock models based on the listed metrics, and the highest performance was demonstrated by the epigenetic clock algorithm based on the PDR metric, which is designed to detect DNA methylation erosion. We show that the performance of WSH-based epigenetic clocks is mildly inferior in accuracy to conventional RRBS-based eAge clocks that are built using average methylation in genomic windows. However, WSH-based clocks only use a few loci in the genome for age prediction. As a result, heterogeneity metrics may have an advantage when RRBS data are used to design a reduced epigenetic clock.

## 2. Results

### 2.1. Development of WSH-Based Regional Blood eAge Clock Models

**Figure 1:**
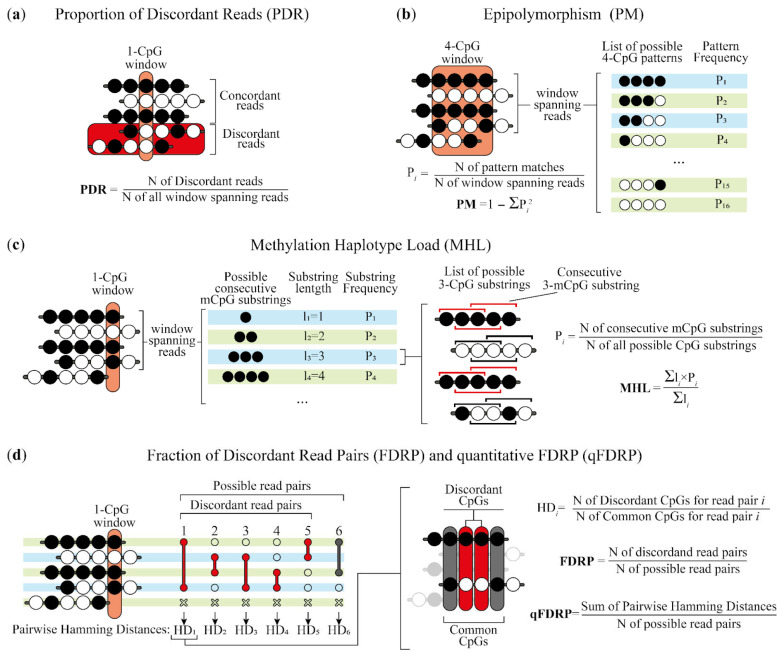
Schematic representation of the procedure of heterogeneity scores calculation using read sequencing data. (**a**) The PDR estimates the prevalence of disordered methylation patterns within CpG spanning reads in the form of the proportion of discordant reads. It classifies each read as discordant if it has both methylated and unmethylated CpGs or if it is concordant otherwise. (**b**) The PM reflects the amount of heterogeneity of DNA methylation for a given genomic region of 4 CpG. The metric is calculated by the frequency of all the possible 4-CpG patterns. (**c**) The MHL is measured by the fraction of fully methylated substrings (mCpG-substrings) of all the possible lengths throughout all the reads spanning the given CpG. It reflects how well the co-methylation haplotypes in a given region are conserved throughout the cell population. (**d**) The FDRP and qFDRP capture within-sample methylation heterogeneity at single CpG resolution. While FDRP calculates the fraction of discordant pairs between all the reads spanning the given CpG, qFDRP estimates the mean Hamming Distance between all the pairs (i.e., the fraction of discordant common CpGs). Note that the Hamming Distance for a concordant read pair is equal to zero.

We used five WSH metrics: MHL (Methylation Haplotype Load), PDR (Proportion of Discordant Reads), PM (Epipolymorphism), FDRP (Fraction of Discordant Read Pairs), and qFDRP (quantitative Fraction of Discordant Read Pairs) (see Figure 1 for an explanation) [10,12,20,21]. Different WSH scores enable the assessment of distinct aspects or the biological phenomena of methylation pattern changes. Intra-molecule score PDR might be considered as a metric of DNA methylation erosion since its elevated values, which are due to stochastic demethylation, have been linked to epigenetic instability in cancer cells [21]. Another intra-molecule score, MHL, captures the homogeneity of co-methylation patterns and serves as an additional indicator of DNA methylation erosion, as it takes on a maximum value of 1 when the region is fully methylated and it is strongly reduced when extended stretches of methylated DNA are disrupted by stochastic demethylation [10]. Inter-molecule score as PM is used to quantify cell-type heterogeneity and describes the DNA methylation patterns in four-CpG windows [20]. FDRP and qFDRP scores are some other metrics that are designed to capture cell-type heterogeneity that analyzes the concordance between the same CpGs within different reads [12]. It should be noted that the basic unit of heterogeneity measurement in the MHL, PDR, FDRP, and qFDRP is a single CpG site, while for the PM, it is four contiguous CpG sites, bringing the different number of analyzed features for each metric. For simplicity, we will hereinafter refer to the listed DNA methylation heterogeneity units as heterogeneity loci regardless of the metric type.

**Figure 2:**
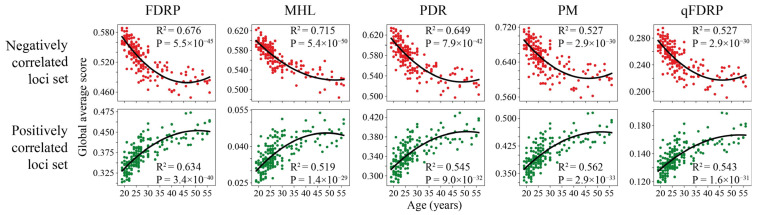
Age dependence of the mean heterogeneity score over positively and negatively age-correlated loci in human blood RRBS datasets. Each point corresponds to the global average score over the entire set of heterogeneity loci per single sample.

In the first step, we evaluated the relevance of WSH scores as an instrument for epigenetic clock design. We analyzed the sequencing data of 182 bisulfite-converted blood DNA samples from donors of different ages (19–56 years, mean age 28.6 years) [23]. We obtained WSH scores for around 2 million CpGs in the case of FDRP and qFDRP scores, well above 1 million CpGs for MHL and PDR, and around 0.7 million stretches comprising four CpGs in the case of PM (Table 1). In order to reduce the diversity of features by age-associated variants, we identified heterogeneity loci with a monotonic relationship between the score value and age within each WSH metric type. Therefore, the scores for each loci were correlated with age using Spearman’s rank correlation coefficient, and the heterogeneity loci that showed a correlation less than |0.25| were filtered out (Table 1). The mean score values for each metric, both positively and negatively correlated, exhibit a quadratic relationship with chronological age (Figure 2), thereby indicating that changes in global heterogeneity occur most rapidly in youth and slow down by old age. It is noteworthy that a nonlinear change in heterogeneity during development was observed in [22], wherein global methylation disorder was investigated in aging mice.

The resulting sets of positively and negatively correlated heterogeneity loci for each WSH score were annotated and associated with genes using ChiPseeker [24]. The total number of genes that overlapped with age-correlated heterogeneity loci is presented in Table S1. Functional annotation and GO term enrichment analysis revealed that genes associated with positively age-correlated heterogeneity loci were enriched in plenty of biological processes, the most significant being body systems development (GO:0048731, GO:0007275, GO:0048856, GO:0032502, GO:0032501), neural tissue development and differentiation (GO:0007399, GO:0022008, GO:0048699, GO:0030182, GO:0048666), and cellular differentiation (GO:0030154) (Table S2). Genes associated with negatively age-correlated heterogeneity loci were significantly enriched in common terms related to organismal development (GO:0048856, GO:0032502, GO:0007275, GO:0007399, GO:0048731), signaling, and regulation of cellular communication (GO:0023051, GO:0010646) (Table S3).

In the next step, we analyzed the heterogeneity loci, demonstrating the strongest correlation with age (|Cor| ≥ 0.5). While the number of highly correlated heterogeneity loci varies from 10 for FDRP to 48 for PDR (Table 1), all of the loci sets are densely within a few genes, fitting into regions of no more than 300 bp in length (Tables S4–S8). Correspondingly, for different WSH scores, the number of associated genes varied between 2 and 6 genes, which often overlapped between metrics (Table 2). Interestingly, some of the listed genes are related to aging or associated with CpGs that are included in different epigenetic age models. For example, CpG sites near the genes *GRM2*, *SCGN,* and *ZIK1* are used in region-based epigenetic clocks, or they are described as age-associated CpGs [25,26,27,28]. *Lin28b* has been found to delay vasculature aging, and *ADRB1* beneficially impacts aging [29,30].

**Figure 3:**
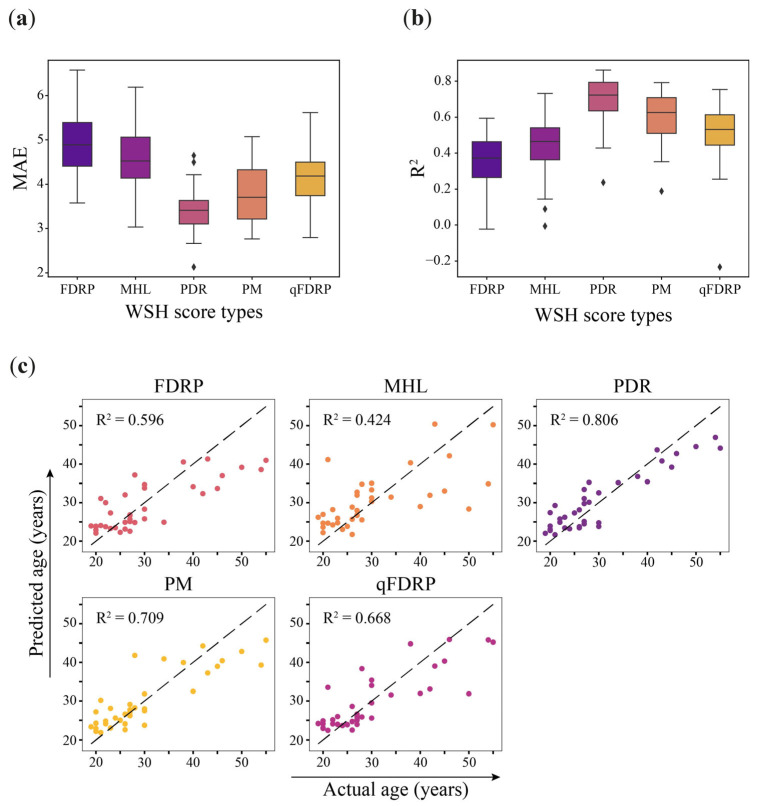
Performance of WSH-based RFR model. (**a**) Observed mean absolute error and (**b**) determination coefficient R^2^ on the training dataset. (**c**) Tested performance of WSH-based eAge models.

Using the corresponding highly correlated heterogeneity loci, a random forest regression model was constructed for each WSH score. Figure 3a,b shows the variances of the models on the training set. The PDR metric shows the best performance (R^2^ = 0.695, MAE = 3.43). The PM and qFDRP metrics show relatively close performance (R^2^ = 0.595 and 0.510, MAE = 3.18, and 4.15, respectively), while the MHL (R^2^ = 0.436, MAE = 4.540) and FDRP (R^2^ = 0.346, MAE = 4.863) performed worse. Next, we evaluated the model on the test samples that were excluded from training and hyperparameter selection. The PDR metric was proven to be the most effective metric (R^2^ = 0.806, MAE = 3.686) (Table 3, Figure 3c). Just as in the case of evaluation on the training set, the FDRP and MHL metrics showed the lowest performance on the test sample.

It is also noteworthy that the sequencing data that we used have reasonably high coverage, while the usual RRBS datasets tend to be of lower quality. To assess the applicability of the WSH scores to detect the dependencies of methylation heterogeneity change with age in RRBS-data samples, we performed a similar analysis to detect the correlations between the heterogeneity loci in RRBS-seq of mesenchymal stem cells samples (32 samples in total, age range of 0–48 years) [31]. Despite the low number of samples and single-end reads sequencing, it can be seen that there are clear associations between age and the heterogeneity scores (Figure S1). Unfortunately, due to the paucity of the samples, we were unable to build the model and assess MAE. However, the ground-age umbilical cord and placenta samples display lower levels of heterogeneity for all of the metrics used, and all of the WSH scores highly correlated with age (R^2^ > 0.8) for both positively and negatively correlated heterogeneity loci.

### 2.2. Assessment of Regional Blood Epigenetic Clock Performance

**Figure 4:**
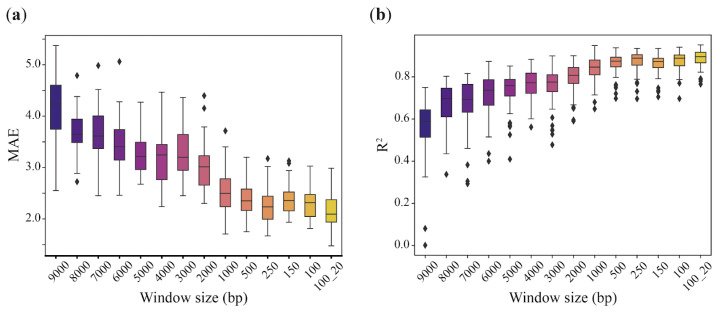
Training performance of regional epigenetic clocks depends on the different genomic windows that were used to calculate average DNA methylation. (**a**) Observed mean absolute error and (**b**) determination coefficient R^2^.

To date, several approaches have been described for constructing epigenetic clock algorithms based on RRBS data, the most productive being the analysis of average methylation over genomic regions of different sizes [13,19]. To compare the efficacy of this regional approach with the heterogeneity-based one, we built an epigenetic clock model that is similar to a previously described method for mice blood RRBS eAge clocks [13]. Briefly, the average methylation frequency over genomic windows of different sizes was calculated, windows containing methylation data were deduplicated, and age-correlated windows were used for further analysis (Table S9). The hyperparameters for LASSO regression were selected on the training set and estimated with the testing set. The performance of the models depending on the window size is shown in Figure 4. It should be noted that, as the window size decreases, the model shows better R^2^ value and MAE. The best prediction accuracy was achieved using a 100 bp sliding window with a 20 bp step size (100_20 in Figure 4 with R^2^ = 0.885 and MAE = 2.164). Usage of windows of smaller sizes (100–1000 bp) performed better, thereby demonstrating R^2^ in the range from 0.837 to 0.874 and MAE ranging from 2.527 to 2.266 (Figure 4, Table 4). Models based on larger genomic intervals (2000–9000 bp) showed an R^2^ that does not exceed 0.8 and an MAE of 3 or more years. The number of regions with non-zero regression coefficients increases with decreasing window size: from 14 for the 9000 bp window to 53 for the 100 bp sliding window.

**Figure 5:**
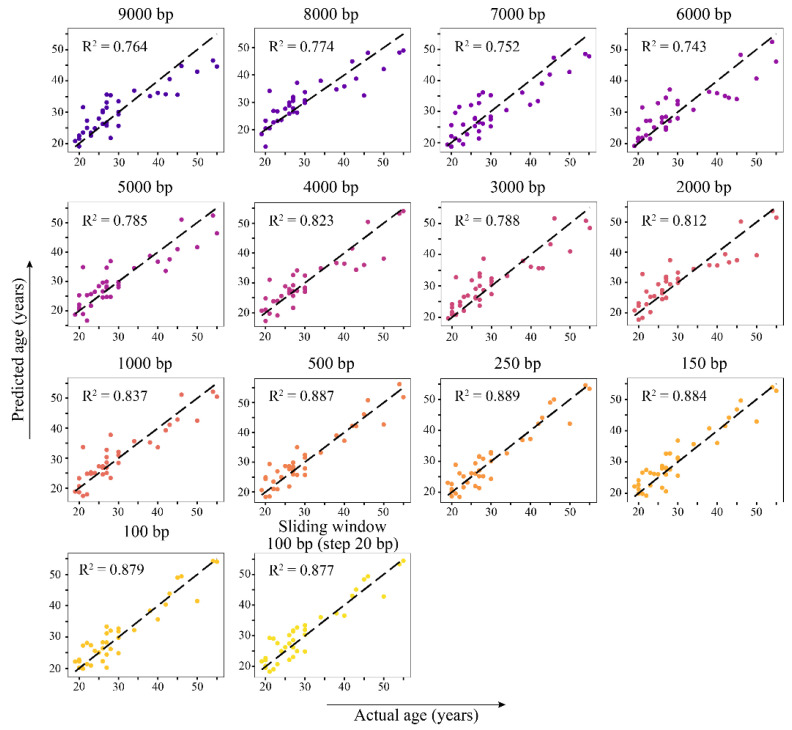
Performance of age prediction using regional epigenetic clocks models on blood data.

The evaluation of the test dataset revealed the best performance of the 250 bp window model, although models built on 100–500 bp windows showed R^2^ above 0.85 and MAE below 3 years (Table 4, Figure 5). Next, the associated genomic windows comprising 250 bp, 150 bp, and 100 bp/sliding windows models yielded a list of 52 genes, with 17 being common to all three genome segmentation approaches (Tables S10–S13). The associated genes are involved in the regulation of apoptosis, control of metabolism, cell division, and differentiation, according to the DAVID database [32].

### 2.3. Regional Blood and WSH-Based Models for Minimized Epigenetic Clocks Design

**Figure 6:**
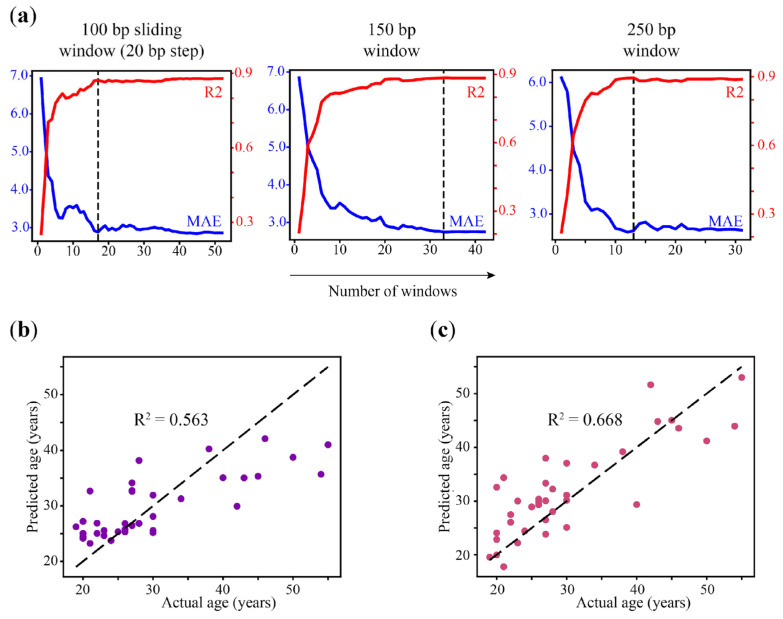
(**a**) Performance of reduced regional eAge models built. Each diagram represents the dependence of R^2^ (red) and MAE (blue) from a number of windows (plotted on the *X*-axis) selected for model evaluation on test data. The minimum number of loci required to build a predictive model without compromising accuracy is indicated by the vertical dashed line. (**b**,**c**) Performance of the age prediction models that were designed using the reciprocal strategy of feature selection. (**b**) The performance of PDR-based clocks built on a set of heterogeneity loci within 100 bp (step 20 bp) sliding windows with age-correlated average methylation. (**c**) Performance of the region-based model built on a set of 100 bp (step 20 bp) sliding windows overlapping 48 highly age-correlated PDR loci.

As mentioned hereinabove, WSH-based epigenetic clock models are based on heterogeneity analysis in just a few genomic regions and, therefore, they might be considered as minimized per se. To estimate the minimum number of windows that can be used to predict age by region-based model without a loss of accuracy, we applied the Recursive Feature Elimination (RFE) method that was implemented in the scikit-learn package. For this purpose, all of the genomic windows with non-zero coefficients in the LASSO regression for 250-bp (32 regions), 150-bp (43 regions), and 100 bp sliding windows (53 regions) models were extracted from each dataset and used to retrain and test the reduced models. We consecutively reduced the number of genomic windows used in each model and tested the accuracy of the resulting eAge models (Figure 6a, Tables S14–S16). We obtained a similar performance of the 250 bp model that was reduced from 32 to 13 windows (R^2^ = 0.889, MAE = 2.633 and R^2^ = 0.896, MAE = 2.618, respectively). The model based on a 150 bp window was reduced up to 33 genomic regions without substantial loss in performance (R^2^ = 0.887, MAE = 2.744). The accuracy of the 100 bp (step 20) sliding window model was conserved until the set of windows was reduced to less than 17 (R^2^ = 0.872, MAE = 2.884) (Table S17). As long as the full 100 bp sliding window model showed the best performance in the initial setup, we analyzed the genes that localized closely to the regions included in the reduced model (Table 5). A few of them were associated with age-associated differentially methylated CpG positions in the blood (*PDCD1LG2*, *NRG2*, *C1orf132*) and with the CpGs included in other epigenetic age estimators (*C1orf132*), while the others were involved in the control of apoptosis, proliferation, and metabolism [33,34,35,36,37,38].

Since the heterogeneity metrics are not directly related to the methylation level, but it does provide complementary information about the methylation pattern at loci, we wanted to test whether age prediction could be improved by combining WSH scores and average methylation by the reciprocal filtering of loci. Therefore, we first modeled heterogeneity-based age prediction using the loci from previously selected 100 bp (step 20 bp) age-correlated sliding windows (Figure 6b, Table 4). Applying heterogeneity metrics only impaired the prediction accuracy despite the fact that filtering by average methylation correlation performed best in a sliding window. We also failed to improve age prediction by generating a 100 bp step 20 sliding window region-based model on regions overlapping 48 previously selected heterogeneity loci with age-correlated PDR metrics, which demonstrated the best performance across WSH scores in the context of age prediction (Figure 6c).

Therefore, the combined approach did not improve the performance of the original models, thereby suggesting that changes in the average methylation level and DNA methylation heterogeneity with aging are not interchangeable in terms of predicting age and might detect different aspects of DNA methylation dynamics. At the same time, it should be noted that the model based on PM and qFDRP metrics shows the best performance in regions where an age-dependent methylation pattern change is observed (Table 6). Since these metrics are designed to capture DNA methylation disorder related to cell-type heterogeneity, this may imply that similar biological causes, at least to some extent, might underlie the heterogeneity changes detected by regional blood epigenetic clocks.

## 3. Discussion

The analysis of RRBS and WGBS data for epigenetic age estimation is associated with a number of technical difficulties, primarily due to the uneven coverage of the genome and the uneven representation of CpG information in different datasets. To date, several models of region-based epigenetic clocks based on the analysis of RRBS data have been proposed [13,19]. For mice, two models of epigenetic blood clocks might be considered the most effective [13]. The first model (regional-based blood clocks, RegBCs) is based on averaged methylation levels from individual CpGs within fixed-size regions that are used for LASSO regression against chronological age [13]. The second one is based on the detection of CpG clusters by the DBSCAN algorithm followed by modeling by LASSO regression (DRegBCs) [13]. Such models outperform the existing analogs based on the analysis of methylation levels of individual CpGs; for example, RegBCs showed a higher correlation with age and lower error (R^2^ = 0.91, MAE = 3.38 months) compared to the previously described blood-specific Pekovich clock (R^2^ = 0.86, MAE = 3.52 months) [13,39].

In our work, an approach similar to the RegBC algorithm was used as a reference, and it demonstrated high accuracy and correlation on human blood samples, achieving R^2^ 0.885 and MAE: 2.164, which is comparable to the most precise blood eAge models based on DNA methylation microarray data with RMSE from 2.04 years [7]. It is noteworthy that RRBS is rarely used for human epigenetic age analysis, e.g., an algorithm called intersection clocks is based on the analysis of individual CpGs, overlapped between the training and testing datasets, which maximizes the use of informative CpG sites but requires training new epigenetic clocks for each dataset [19].

The motivation for our work was to assess changes in the DNA methylation heterogeneity during aging and the applicability of such a marker for estimating age and constructing epigenetic clocks. Indeed, as mentioned above, reproducible changes in the methylation patterns are observed during aging, which might be associated with changes in the cellular composition of organs and tissues, changes in the functional state of cells, and dysfunction of DNA methylation maintenance systems [2,16,17,18,21,40]. Despite the lack of consensus on the physiological causes of aging that the epigenetic clock detects, a plethora of works evaluate stochastic changes in the methylation pattern, which is also referred to as epigenetic drift in the context of aging [18,41,42]. Therefore, metrics assessing DNA methylation disorder and heterogeneity, such as regional disorder and regional entropy, have been successfully used to predict age and assess the influence of common lifespan manipulation, development, and cellular dedifferentiation on epigenetic age estimates in mice [22].

Using five different WSH scores, we constructed WSH-based eAge clocks models. Interestingly, the best performance was shown by epigenetic clocks based on the PDR score, which is designed to capture methylation erosion rather than cellular heterogeneity. This finding, to some extent, echoes the observation that variability in blood cellular composition is unable to fully account for the detected methylation drift [41]. However, all heterogeneity metrics revealed disordered regions associated with genes that are related to aging or associated with CpGs that are included in different epigenetic age models. Our results show that the epigenetic clock model based on the analysis of average methylation within selected regions showed better performance than WSH-based eAge models. However, it is important to note that the minimized region-based eAge clock model requires the analysis of at least 13 genomic regions, while WSH-eAge models are based on the analysis of a smaller number of genomic regions of comparable size. It is conceivable that the WSH-eAge modeling may be the preferred approach for the design of minimized epigenetic clocks analyzed by targeted BS-seq. This requires experimental validation, but the accuracy that we have modeled is potentially consistent with the most accurate minimized eAge blood clocks that are currently available, demonstrating the error in the range from 3.5 to 8.8 years [7]. It is important to mention that WSH-eAge modeling requires relatively high genomic coverage of the regions to allow WSH score calculation; therefore, it should be applied for deep-sequenced samples or targeted bisulfite sequencing data. Another possible issue in the usage of WSH-eAge models might arise from uneven coverage of RRBS data in separate experiments, which could require retraining of the model based on the genomic regions that are sequenced in all datasets, e.g., in a manner similar to the described algorithm of intersection clock [19].

## 4. Materials and Methods

### 4.1. Source Data

Bisulfite sequencing data of 182 human blood samples were downloaded from the ENA database via BioProject identifier PRJNA531784 [23]. In addition, a dataset containing 32 RRBS samples from mesenchymal stem cells was downloaded via BioProject identifier PRJNA349025 [31].

### 4.2. Data Processing and WSH Scores Calculation

Each sample was treated as follows. In the first step, adapter removal and quality control of short paired-end reads were performed using TrimGalore! v0.6.10 with -rrbs option [43]. The processed reads were then aligned on a GRCh38 (hg38) reference genome assembly using Bismark v0.24.2 with Bowtie 2 v2.5.2 [44]. The resulting BAM alignments were sorted by coordinate using SAMtools v1.6 [45]. The sorted BAM files were then used for the calculation of methylation heterogeneity metrics using Metheor v0.1.8 with the default parameters [46]. Overall, five metrics of heterogeneity (PM, FDRP, MHL, PDR, qFDRP) were calculated for each sample independently. The coverage files produced by Bismark, which provide information about the level of methylation for each CpG in a sample, were used for the calculation of average CpG methylation.

### 4.3. Heterogeneity Loci Processing and Annotation

To identify the age-related heterogeneity loci within the blood samples, Spearman’s rank correlation between the given heterogeneity score and donor age, along with the FDR-adjusted *p*-value, was calculated for each loci using a custom script. Next, within each score type, the loci were divided into three subsets: (1) loci with a negative correlation (cor ≤ −0.25, Padj < 0.05), (2) loci with a positive correlation (cor ≥ 0.25, Padj < 0.05), and (3) with a modulus correlation above 0.5 (|cor| ≥ 0.5, Padj < 0.05). Each subset of loci was then annotated using ChiPseeker (distance to TSS = 0) and gProfiler, and subset (3) was used for building a score-specific Random Forest Regression (RFR) model [24,47].

### 4.4. WSH-Based Epigenetic Clock Construction Using Random Forest Regression

RFR modeling was performed using the scikit-learn v1.4.0 Python package [48]. In this analysis, the dataset was divided into a training (80%) and a test (20%) set of samples. The hyperparameters were selected through five-fold cross-validation on the training samples. The best hyperparameters were used to evaluate the stability and reliability of the model through five iterations of ten-fold cross-validation. In the last stem, the model was evaluated on the test set, which did not undergo the training process.

### 4.5. Genome Segmentation and Calculation of Average Methylation Level

For a window-based estimation of CpG methylation, all CpG positions with coverage of less than five were filtered out. Next, the proportion of methylated CpGs in selected sets of genome intervals was calculated for each sample using a custom script. For these purposes, 14 sets of intervals were produced. To obtain the coordinates for each set, the genome (excluding X, Y, and M) was divided into consecutive fragments in different ways: using equal-sized bins of 100 bp–9 kb or using a sliding window algorithm (100 bp intervals with 20 bp step). The resulting BED formatted tables with the same interval set were combined by coordinates. All of the intervals without any CpG or with undetermined average methylation in at least one sample were filtered out.

To reduce the number of analyzed interval features in average methylation datasets, the Pearson correlation coefficient was calculated between the average methylation and the age value for each interval. Intervals were filtered if their absolute correlation value was less than 0.5 (Padj < 0.05). For the interval set, which was produced with a sliding window algorithm, deduplication was also performed to remove of identical rows. Produced data tables were then used for the LASSO regression modeling of age.

### 4.6. Region-Based Epigenetic Clock Construction Using LASSO Regression

To build an age prediction model on the genomic interval-based average methylation data, LASSO regression was chosen. The model was built using the scikit-learn v1.4.0 package in Python [48]. Each dataset was divided into a training set (80%) and a test set (20%). The selection of the alpha hyperparameter was carried out through ten-fold cross-validation on the training set. Next, the best hyperparameter alpha was used to build and evaluate the model on the training dataset and for evaluation on the test set.

## Figures and Tables

**Table 1:** The diversity of heterogeneity loci for each metric in the dataset of donor blood samples. For each metric, the number of loci with a selected Spearman’s correlation is also indicated.

Heterogeneity Metrics	Number of Heterogeneity Loci	Cor ≤ −0.25	Cor ≥ 0.25	|Cor| ≥ 0.5
FDRP	1,918,416	2311	16,108	10
MHL	1,287,083	934	40,393	22
PDR	1,291,638	3000	25,675	48
PM	696,871	2297	11,478	20
qFDRP	1,918,416	3548	34,856	27

**Table 2:** List of genes associated with highly correlated heterogeneity loci in all WSH scores.

Metric	Ensembl Gene ID	Gene Symbol	Gene Name	Biological Process	Gene Regions
MHL	ENSG00000164082	*GRM2*	glutamate metabotropic receptor 2	negative regulation of adenylate cyclase activity	Promoter; 5’ UTR; Exon (1 of 4); Intron (1 of 5)
	ENSG00000158815	*FGF17*	fibroblast growth factor 17	positive regulation of protein phosphorylation	Exon (5 of 5)
PDR	ENSG00000043591	*ADRB1*	adrenoreceptor beta 1	positive regulation of heart rate by epinephrine-norepinephrine	Exon (1 of 1)
	ENSG00000170549	*IRX1*	Iroquois homeobox 1	negative regulation of transcription from RNA polymerase II promoter	Exon (2 of 4)
	ENSG00000122584	*NXPH1*	neurexophilin 1	NA	Intron (2 of 2)
	ENSG00000079689	*SCGN*	secretagogin, EF-hand calcium-binding protein	regulation of cytosolic calcium ion concentration	5’ UTR; Exon (1 of 10)
	ENSG00000212719	*LINC02693*	long intergenic non-protein coding RNA 2693	NA	Intron (2 of 6)
	ENSG00000171649	*ZIK1*	zinc finger protein interacting with K protein	regulation of transcription from RNA polymerase II promoter	5’ UTR
PM	ENSG00000150594	*ADRA2A*	adrenoreceptor alpha 2A	positive regulation of cytokine production	Exon (1 of 1)
	ENSG00000043591	*ADRB1*	adrenoreceptor beta 1	positive regulation of heart rate by epinephrine-norepinephrine	Exon (1 of 1)
	ENSG00000164082	*GRM2*	glutamate metabotropic receptor	negative regulation of adenylate cyclase activity	Promoter; Exon (1 of 4); Intron (1 of 5)
	ENSG00000212719	*LINC02693*	long intergenic non-protein coding RNA 2693	NA	Intron (2 of 6)
	ENSG00000122584	*NXPH1*	neurexophilin 1	NA	Intron (2 of 2)
FDPR	ENSG00000164082	*GRM2*	glutamate metabotropic receptor	negative regulation of adenylate cyclase activity	5’ UTR; Exon (1 of 4); Intron (1 of 5)
	ENSG00000164093	*PITX2*	paired-like homeodomain	negative regulation of transcription from RNA polymerase II promoter	Exon (3 of 5)
qFDPR	ENSG00000043591	*ADRB1*	adrenoreceptor beta 1	positive regulation of heart rate by epinephrine-norepinephrine	Exon (1 of 1)
	ENSG00000164082	*GRM2*	glutamate metabotropic receptor	negative regulation of adenylate cyclase activity	Promoter; 5’ UTR; Exon (1 of 4); Intron (1 of 5)
	ENSG00000187772	*LIN28B*	lin-28 homolog	miRNA catabolic process	5’ UTR
	ENSG00000212719	*LINC02693*	long intergenic non-protein coding RNA 2693	NA	Intron (2 of 6)
	ENSG00000164093	*PITX2*	paired-like homeodomain	negative regulation of transcription from RNA polymerase II promoter	Exon (3 of 5)
	ENSG00000269897	*COMMD3-BMI1*	COMMD3-BMI1	sodium ion transport	Distal Intergenic

**Table 3:** Performance of WSH-based eAge models estimated on the test dataset.

Metrics	Test Sample	
	R^2^	MAE
FDRP	0.596	4.929
MHL	0.424	5.334
PDR	0.806	3.686
PM	0.709	3.969
qFDRP	0.668	4.278

**Table 4:** Performance of the regional epigenetic clocks models on the blood dataset.

Window Size	Cross Validation		Test Sample		Number of Regions with Non-Zero Coefficient in Regression Model
	R^2^	MAE	R^2^	MAE	
9000 bp	0.556	4.192	0.764	3.814	14
8000 bp	0.665	3.698	0.774	3.504	16
7000 bp	0.668	3.671	0.752	4.096	12
6000 bp	0.718	3.437	0.743	3.889	13
5000 bp	0.735	3.288	0.785	3.416	18
4000 bp	0.760	3.187	0.823	3.043	13
3000 bp	0.756	3.273	0.788	3.460	17
2000 bp	0.791	3.019	0.812	3.305	16
1000 bp	0.837	2.527	0.837	3.052	20
500 bp	0.862	2.392	0.887	2.713	28
250 bp	0.873	2.266	0.889	2.633	32
150 bp	0.858	2.379	0.884	2.750	43
100 bp	0.874	2.299	0.879	2.712	42
100 bp sliding window (20 bp step size)	0.885	2.164	0.877	2.866	53

**Table 5:** List of genes that were associated with minimized 100 bp/sliding window regional eAge model.

Ensembl Gene ID	Gene Symbol	Gene Name	Biological Process
ENSG00000114738	*MAPKAPK3*	MAPK-activated protein kinase 3	MAPK cascade
ENSG00000197142	*ACSL5*	acyl-CoA synthetase long-chain family member 5	long-chain fatty acid metabolic process
ENSG00000164082	*GRM2*	glutamate metabotropic receptor 2	negative regulation of adenylate cyclase activity
ENSG00000148734	*NRFFR1*	neuropeptide FF receptor 1	G-protein coupled receptor signaling pathway
ENSG00000115594	*IL1R1*	interleukin 1 receptor type 1	inflammatory response
ENSG00000142235	*LMTK3*	lemur tyrosine kinase 3	protein phosphorylation
ENSG00000158458	*NRG2*	neuregulin 2	signal transduction
ENSG00000010322	*NISCH*	nischarin	apoptotic process
ENSG00000197646	*PDCD1LG2*	programmed cell death 1 ligand 2	adaptive immune response
ENSG00000106772	*PRUNE2*	prune homolog 2 with BCH domain	apoptotic process
ENSG00000163239	*TDRD10*	Tudor domain containing 10	P-granule organization
ENSG00000165626	*BEND7*	BEN Domain-Containing Protein 7	
ENSG00000203709	*MIR29B2CHG/C1orf132*	MIR29B2 And MIR29C Host Gene	gene silencing by miRNA
ENSG00000236333	*TRHDE-AS1*	TRHDE antisense RNA 1	
ENSG00000166135	*HIF1AN*	hypoxia-inducible factor 1 subunit alpha inhibitor	peptidyl-histidine hydroxylation
ENSG00000180720	*CHRM4*	cholinergic receptor muscarinic 4	carbohydrate metabolic process
ENSG00000164197	*RNF180*	ring finger protein 180	protein polyubiquitination

**Table 6:** The performance of WSH-based models built on genomic windows comprising 100 bp/sliding window regional eAge model.

Metrics	Test Sample	
	R^2^	MAE
FDRP	0.635	4.662
MHL	0.648	4.444
PDR	0.563	4.993
PM	0.697	4.293
qFDRP	0.722	3.962

## Data Availability

The data that support the findings of this study are openly available in the European Nucleotide Archive (Accessions: PRJNA531784, PRJNA349025).

## Supplementary Materials

The following supporting information can be downloaded at https://www.mdpi.com/article/10.3390/ijms25094967/s1.
