# Supplementary material for: Age Prediction Using DNA Methylation Heterogeneity Metrics
Source: Int J Mol Sci. 2024 May 2;25(9):4967. doi: 10.3390/ijms25094967 (PMC11084170; doi:10.3390/ijms25094967)
Supplement: Supplementary file 1 [file ijms-25-04967-s001.zip › Figure S1_with caption.pdf]

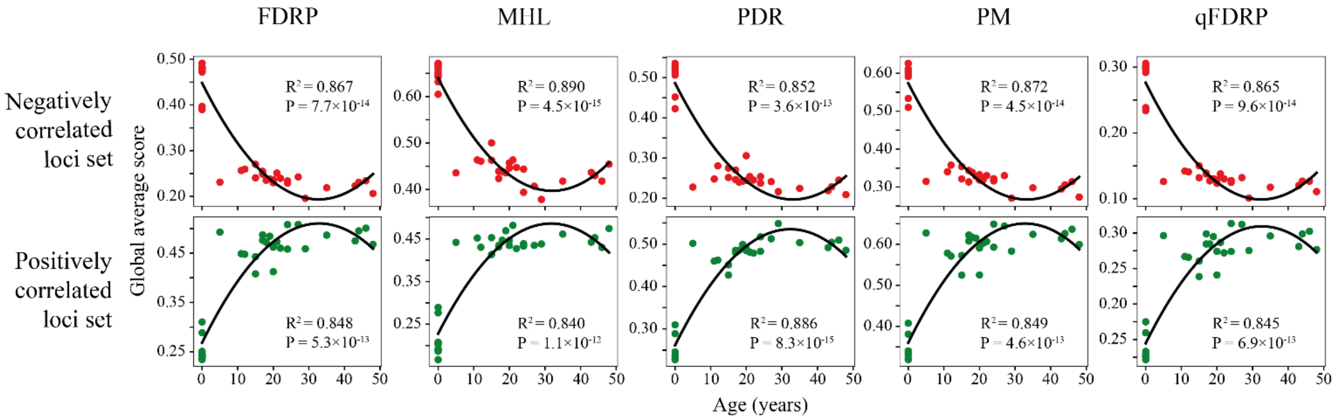

Figure S1. Age-dependent dynamics of the mean heterogeneity score over sets of loci with positively or negatively age-correlated metric values based on MSC RRBS datasets. Each point corresponds to the global average score over the entire set of heterogeneity loci per single sample.
