# Supplementary figures and images for: Age Prediction Using DNA Methylation Heterogeneity Metrics
Source: Int J Mol Sci. 2024 May 2;25(9):4967. doi: 10.3390/ijms25094967 (PMC11084170; doi:10.3390/ijms25094967)

Negatively  
correlated  
loci set

FDRP

MHL

PDR

PM

qFDRP

Positively  
correlated  
loci set

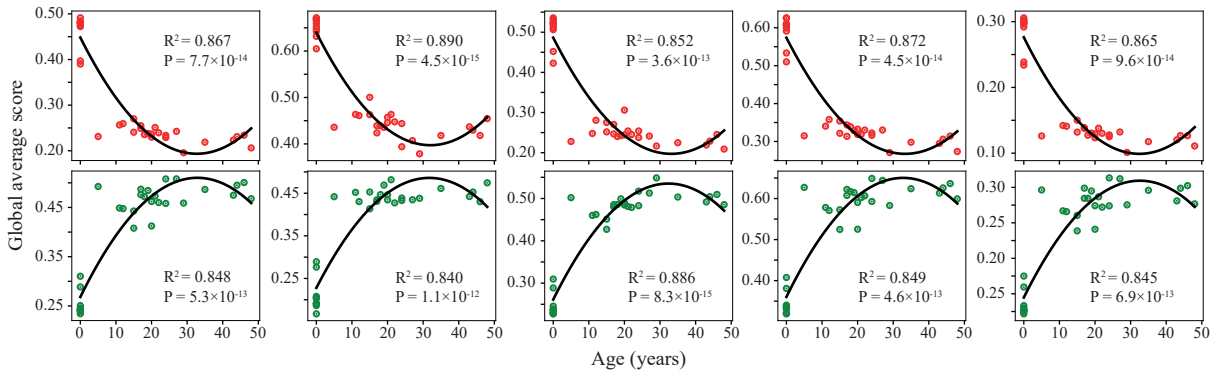

Supplement: Supplementary file 1 [file ijms-25-04967-s001.zip › Figure S1.pdf]

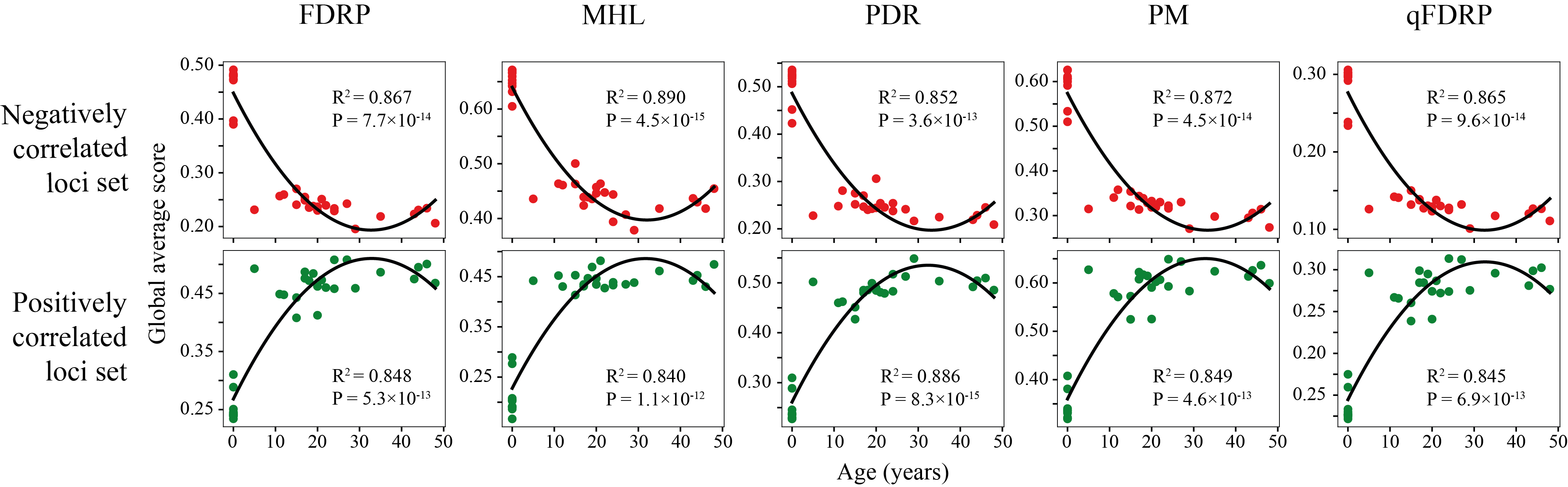

Supplement: Supplementary file 1 [file ijms-25-04967-s001.zip › Figure S1.png]
